# Representations of Abstract Relations in Infancy

**DOI:** 10.1162/opmi_a_00068

**Published:** 2022-12-16

**Authors:** Jean-Rémy Hochmann

**Affiliations:** CNRS UMR5229 - Institut des Sciences Cognitives Marc Jeannerod, 67 Boulevard Pinel, 69675, Bron, France; Université Lyon 1 Claude Bernard, France

**Keywords:** abstract relations, infants, language of thought

## Abstract

Abstract relations are considered the pinnacle of human cognition, allowing for analogical and logical reasoning, and possibly setting humans apart from other animal species. Recent experimental evidence showed that infants are capable of representing the abstract relations *same* and *different*, prompting the question of the format of such representations. In a propositional language of thought, abstract relations would be represented in the form of discrete symbols. Is this format available to pre-lexical infants? We report six experiments (*N* = 192) relying on pupillometry and investigating *how* preverbal 10- to 12-month-old infants represent the relation *same*. We found that infants’ ability to represent the relation *same* is impacted by the number of individual entities taking part in the relation. Infants could represent that four syllables were the same and generalized that relation to novel sequences (Experiments 1 and 4). However, they failed to generalize the relation *same* when it involved 5 or 6 syllables (Experiments 2–3), showing that infants’ representation of the relation *same* is constrained by the limits of working memory capacity. Infants also failed to form a representation equivalent to *all the same*, which could apply to a varying number of same syllables (Experiments 5–6). These results highlight important discontinuities along cognitive development. Contrary to adults, preverbal infants lack a discrete symbol for the relation *same*, and rather build a representation of the relation by assembling symbols for individual entities.

## INTRODUCTION

How do young infants think, before they have words to express their thoughts? Thoughts can be characterized in terms of content (what they are about) and format (what they are like). Of course, the two issues are not independent; certain format favour certain content, and certain content may require certain format. The present work aims at improving our characterization of infants’ thought format, focusing on the case of abstract relations.

A discussion about the format of mental representations typically begins with the opposition between perceptual-like, iconic images and language-like, symbolic propositional representations (Paivio, 1971; Bruner, 1964; Fodor, 1975, 2008). While Piaget (1954) thought young infants initially lack mental representations altogether, the idea that infants possess perceptual or perceptual-like mental representations is not controversial anymore. Such representations can account for object recognition (Wilcox & Baillargeon, 1998), visuospatial operations such as mental rotation (Moore & Johnson, 2008, 2011; Quinn & Liben, 2008, 2014) and mental simulation (Téglás et al., 2011). Some researchers have even proposed that, early in life, all of mental representations consist in “impoverished perceptual-like images” (Bruner, 1964; Mandler, 1992; Mandler & Cánovas, 2014).

Do infants also possess a propositional *language of thought* (Fodor, 1975; Macnamara, 1972; Pylyshyn, 1973)? Components of such language of thought are discrete abstract symbols referring to entities and their relations, which are combined to form propositions, much like words are combined to form sentences. Discrete symbols that enter propositional representations are thus particularly efficient to represent abstract relations (Hochmann & Papeo, 2021; Premack, 1983). Evidence that infants possess abstract concepts (Carey, 2009) including abstract relations (Hochmann, 2021) and precursors of logical operators (Cesana-Arlotti, Kovács & Téglás, 2020b; Hochmann & Toro, 2021) could indicate that infants possess propositional representations, but alternative accounts of these data remain possible (Leahy & Carey, 2020; Hochmann, 2020).

To progress in our understanding of what it is like to think without words, as an infant, we sought to characterize the format of the representation of an abstract relation, the relation *same*. Results show that infants are capable of representing *same* and suggest that the format of that representation is inherently different from the adults’, challenging the view that infants possess a propositional language of thought.

### The Relation *Same*

Children learn the words “same” and “different”, as applied to pairs of individuals (e.g., △ and △ are the same; △ and ◻ are different), in the fourth year of life (Christie & Gentner, 2014; Hochmann et al., 2017; Hochmann et al., submitted). However, there is now substantial evidence that even young infants are already able to represent the relation *same* between two individuals (see Hochmann, 2021 for review). Neonates exhibit a specific neural response to sequences of syllables containing two identical syllables (Gervain et al., 2008, 2012). Infants as young as 3 months habituate to exemplars of the relation *same* (Addyman & Mareschal, 2010; Anderson et al., 2018; Ferry et al., 2015; Tyrrell et al., 1991) and 6- to 18-month-olds can condition behavioral responses to the perception of two identical stimuli (Hochmann et al., 2011, 2016, 2018b; Kovács, 2014; Walker & Gopnik, 2014, 2017).

Going beyond the question of whether infants represent the relation same, in the present work, we ask *how* infants represent such relation. In the studies reviewed above, infants generalized the conditioned response to novel pairs of items that instantiated the relation *same*. Evidence for generalization denotes abstraction, suggesting that infants’ representation of *same* cannot be reduced to a perceptual-like image (Premack, 1983). Does generalization also imply that, like adults, infants represent the relation *same* with a discrete symbol dissociated from the representation of entities? Not necessarily.

Extensive research suggests that the representation of the relation *same* might not be adult-like before 4–5 years of age. While infants succeed in a simple match-to-sample task (e.g., matching △ to △) (Hochmann et al., 2016), children below 4 or 5 years fail in a relational match-to-sample task (RMTS), where they have to match pairs of stimuli that instantiate the relations *same* or *different* (e.g., △ △ matches ◻ ◻; △ ◯ matches ◻ X) (Premack, 1983; Hochmann et al., 2017). Despite a handful of successes by parrots, crows and highly-trained monkeys (see Smirnova et al., 2021 for review), this task also remains extremely difficult for non-human animal species (Flemming & Thompson, 2021; Gentner et al., 2021). Moreover, children younger than 4 and animals do not spontaneously categorize arrays of 16 pictures as *all the same* vs. *not all the same* or *all the same* vs. *all different*, whereas older children and adults do (Hochmann et al., 2017; Fagot et al., 2001). Those failures suggest that the infants’ (and animal) representation of the relation *same* differs from that of older children and adults. It has been proposed that the acquisition of the word “same” plays a role in the transition between the two types of representation. Even though a causal link between these abilities has yet to be demonstrated, those children who understand the word “same” or spontaneously produce the words “same” and “different” tend to succeed in RMTS and to categorize arrays as *all the same* vs. *not all the same* (Christie & Gentner, 2014; Hochmann et al., 2017). Similarly, among great apes, those who have acquired symbols for the relations *same* and *different* perform better in RMTS (Premack, 1983, Thompson et al., 1997). In sum, children may not possess an adult-like representation of *same* before they acquire a symbol like the word “same”^1^.

We are facing an apparent paradox: infants succeed in a number of tasks that necessitate recognizing the abstract relation *same* in different pairs of stimuli (e.g., Hochmann et al., 2018b), but they fail at matching two pairs instantiating *same* in RMTS (Christie & Gentner, 2014; Hochmann et al., 2017). To account for these data, we propose that, while abstraction is sufficient to succeed in match-to-sample, habituation and conditioned discrimination tasks, the RMTS additionally requires that the representation of abstract relations be discrete, i.e., distinct and fully dissociated from the representation of the entities involved in the relation. Acquiring an external symbol such as the word “same” may trigger or facilitate the construction of such discrete representation (Premack, 1983).

Instead of a discrete symbol for the relation, we propose that the infants’ representation of *same* can consist of a repeated variable (Hochmann et al., 2016; Marcus et al., 1999). If *X* is a variable defined in the domain Σ, two same entities in that domain can be represented as *X* ∈ Σ, (*X X*). Importantly, in this format of representation, there is no discrete symbol for the relation but one symbol (one instance of the same variable) for each individual entity. Thus, when infants learn a rule exemplified with two cups, two balls, two ducks, etc., they would represent (*X X*), which means two same objects. The domain of the variable *X* defines the domain where the relation applies: if *X* belongs to the domain of shapes, (*X X*) means *two same shapes*; if *X* belongs to the domain of colors, (*X X*) means *two same colors*; and so on. Furthermore, the representation of the relation *same* is tight to a specific number of entities: (*X X*) is a representation of *two-same*, (*X X X*) is a representation of *three-same*, and so on. In fact, in this format of representation, not only isn’t there a discrete symbol for the relation, but the relation, the domain or dimensions to which it applies, and the numerosity are all intertwined.

This format of representation relying on variables is abstract, affording the generalization of the relation *same*, but lacks a discrete symbol *S* that is dissociated from the representation of the entities in the relation and could be integrated in a propositional language of thought to generate strings such as *X S Y* (or *S*(*X*, *Y*)) meaning *X is the same as Y*. The format that we propose for the infants’ and young children’s representation of *same* can account both for the success in habituation and conditioned discrimination tasks (e.g., Hochmann et al., 2018b), and for the failure in RMTS (Christie & Gentner, 2014; Hochmann et al., 2017). Indeed, *X* being a variable, its value can change over time; it can refer successively to various objects, e.g., *a duck*, *a cup*, etc. Infants can thus use (*X X*) to represent successively (*duck duck*) and (*cup cup*). Consequently, they can habituate to (*X X*) (e.g., Anderson et al., 2018) and learn to condition a response to (*X X*) (e.g., Hochmann et al., 2018b). But *X* can be assigned only one value at a given time; it cannot refer simultaneously to different objects such as a duck and a cup. In consequence, children cannot use the (*X X*) representation to match (*duck duck*) and (*cup cup*), as is required to succeed in RMTS. Success in RMTS may require, or be facilitated by, the acquisition of a discrete symbol *S* for the relation *same*, in order to relate both (*duck duck*) and (*cup cup*) to *S*, and subsequently match *S* to *S*.

The format of representation that we propose may also account for infants’ and young children’s difficulty to represent *all the same* (Hochmann et al., 2017). Indeed, *all the same* applies equally to arrays of varying numbers of entities. With respect to quantification, *all the same* thus requires disregarding the number of elements involved in the relation to focus on another property: exhaustivity. Representations of, for instance, *two-same* as (*X X*) and *three-same* as (*X X X*) impede this process as each of these schemas is tight to a specific numerosity, implicitly represented by the number of individual instances of *X*. For *all* or exhaustivity to apply to a representation of *same*, that representation must be first segregated from the representation of numerosity. The observed association between spontaneous categorization of arrays as *all the same* vs. *not all the same* and the use of the word “same” in childhood (Hochmann et al., 2017) further suggests that representing *all the same* requires a discrete symbol for the relation *same*, which is not only segregated from the representation of entities, but also from the representation of numerosity.

### Current Study

Here, we tested the hypothesis that pre-lexical infants represent the relation *same* with the format (*X X*), i.e., with an abstract symbol for each entity to which the relation *same* applies, but no discrete symbol for their relation. We reasoned that, if this hypothesis is correct, the number of same elements that can be represented as *same* should be constrained by the limits of the working memory capacity. Furthermore, because the representation of *same* that we propose is tight to a specific numerosity, representing *all the same*, hence disregarding the number of same entities, should be difficult.

Working memory is a limited short-term memory system that maintains information accessible for cognitive operations. In adults, working memory capacity is generally limited to about four items (Cowan, 2001), with some variability depending on what is being represented (Brady et al., 2016) and on interferences between individual representations (Endress & Potter, 2014; Schurgin et al., 2020). Research on infants’ working memory suggests that at 11 months, the working memory capacity is about three or four items (Benavides-Varela & Reoyo-Serrano, 2021; Feigenson & Carey, 2003; Feigenson et al., 2002; Ross-Sheehy et al., 2003). As previous research found that 11-month-old infants could represent the relation *same* between 4 syllables (Hochmann & Toro, 2021), we expected four items (i.e., syllables) to be the limit beyond which 11 months infants would fail to apply the relation *same*.

In Experiments 1 and 4, we asked whether infants can represent the relation *same* between four entities. In Experiments 2–3, we asked whether infants can represent the relation *same* between a number of individuals that exceeds the alleged working memory capacity (five or six). Finally, we asked whether infants can ignore the number of entities involved in the relation *same*, effectively representing that *all* items are the same, whether there are three, four or five of them (Experiment 5), or whether there are two, three or four of them (Experiment 6). In each experiment, 10- to 12-month-old infants were exposed to series of sequences of syllables. Crucially, even 12-month-olds are still years away from producing or understanding the word “same” (Hochmann et al., 2017; Hochmann et al., submitted). We used syllables, which are well represented, discriminated and categorized by the end of the first year of life (Hochmann & Papeo, 2014; Werker & Tees, 1984). The syllables varied from one trial to another (48 different syllables were used for each infant). In the Unbalanced group, most sequences (75%) matched a structure defined by the relation *same* (Same sequences; e.g., AAAA in Experiment 1: *ba ba ba ba*, *ko ko ko ko*, *mi mi mi mi*). If infants were able to represent the Same structure, they should come to expect all sequences to be composed of identical syllables, and should be surprised by the insertion of a different syllable in 25% of sequences containing one different syllable (the Different sequences; e.g., AAAB: *nu nu nu la*). The violation of expectation was measured with pupillometry. Increase in arousal, attention or cognitive load triggers an increase of pupil diameters (Beatty & Kahneman, 1966; Hess & Polt, 1960; Laeng et al., 2012). Relevant to the present study, in oddball paradigms, pupils dilate in reaction to rare auditory stimuli in both adults (Qiyuan et al., 1985; Quirins et al., 2018) and infants (Hochmann & Papeo, 2014; Hochmann & Toro, 2021), most likely as a result of increased attention or arousal due to an unexpected event.

Different sequences could be surprising because they violate the expected Same-sequence in their global relational structure (e.g., AAAB, rather than AAAA), but also because they contain a local syllable change (from A to B). Furthermore, we may expect the effect of a local change to increase with the number of repetitions of the same syllable (e.g., a stronger effect is expected for AAAAAB sequences than for AAAB sequences). To disentangle the “global effect”, related to the violation of the relational structure, from the effect of the local change, each experiment included a Control group, for whom 50% of sequences followed the Same structure and 50% followed the Different structure. The effect of the local change is by definition independent from the frequency of the Different structure, and is therefore identical in the Unbalanced and Control groups. The global effect, in contrast, should only be observed in the Unbalanced groups.

## MATERIAL AND METHODS

### Participants

One-hundred-ninety-two infants (age range: 9 m 28 d – 12 m 21 d; average: 10 m 30 d) participated in Experiments 1–6 (see Table 1 for detailed information). Fourteen additional infants were tested but excluded for not providing a sufficient number of trials. Thirty-two infants participated in each experiment. Half of them were included in the unbalanced group and half in the control group. The sample size of 16 participants per group was chosen following Hochmann and Toro (2021, Experiment 1), after a power analysis using G*Power (Faul et al., 2009) showed this sample size to be higher than the minimal required sample size of 13 participants for *α* = .05, 1 − *β* = .80, and *d* = .74. All participants were recruited through the consultation of birth records at the city halls. Infants were tested in the babylab of Institut des Sciences Cognitives Marc Jeannerod in Bron, France. Parents received travel reimbursement and gave informed consent before participation. The study was approved by the local ethics committee (CPP sud-est II).

**Table 1.** Demographic information

**Experiment**	**Group**	**Age range**	**Mean age**	**N female**
Experiment 1	Unbalanced	10 m 6 d – 12 m 15 d	11 m 6 d	9
	Control	10 m 4 d – 11 m 26 d	10 m 26 d	7
Experiment 2	Unbalanced	10 m 12 d – 11 m 30 d	10 m 23 d	5
	Control	10 m 3 d – 12 m 0 d	10 m 25 d	10
Experiment 3	Unbalanced	10 m 8 d – 12 m 21 d	11 m 8 d	7
	Control	10 m 14 d – 12 m 4 d	11 m 6 d	5
Experiment 4	Unbalanced	10 m 3 d – 11 m 28 d	10 m 25 d	7
	Control	10 m 13 d – 11 m 24 d	11 m 1 d	6
Experiment 5	Unbalanced	10 m 1 d – 12 m 7 d	11 m 6 d	6
	Control	9 m 28 d – 12 m 3 d	10 m 27 d	9
Experiment 6	Unbalanced	10 m 6 d – 11 m 17 d	10 m 21 d	10
	Control	10 m 9 d – 11 m 30 d	11 m 2 d	6

### Stimuli

As previously described (Hochmann & Toro, 2021), 48 syllables were created with the artificial speech synthesizer MBROLA (French voice database FR4), with phoneme duration of 120 ms and pitch of 200 Hz. We used 12 consonants (/b/, /d/, /g/, /p/, /t/, /k/, /v/, /f/, /s/, /l/, /m/, /n/) and 4 vowels (/a/, /i/, /o/, /u/). Each syllable was normalized to an intensity of 70 dB. The video shown repeatedly on the screen of the eyetracker consisted in an animated video clip showing a smiling cartoon character jumping repeatedly. The movements in this video clip were not synchronized to the presentation of the acoustic stimuli.

### Procedure

As previously described (Hochmann & Toro, 2021), infants sat on their parent’s laps in front of a Tobii eyetracker T60XL. The presentation of stimuli and the recording of eye-tracking data was controlled by PsyScope X (https://psy.cns.sissa.it). All lights in the room were switched off, except for those coming from the eyetracker screen. Each trial started automatically when infants fixated a central blinking cross presented on the screen. The central blinking cross disappeared and the jumping character appeared at the center of the screen. The pupil size is particularly sensitive to variations of luminance. To ensure that any observed effect could not be attributed to variations of luminance, contrast or any other visual features of the stimuli, the exact same video was shown in all trials. The first syllable began 200 ms after the onset of the video clip. The onsets of two successive syllables were separated by 500 ms. One sequence of syllables was played in each trial. All trials had the same duration: 5217 ms.

Experiments 1–3 presented below were planned. Experiments 4–6 were added after observing the results of Experiments 1–3.

*Experiment 1*: Half of the participants were included in the unbalanced group. The first 8 trials consisted in standard Same sequences, respecting the AAAA structure. For the rest of the experiment, 75% of trials were standard Same sequences (AAAA; e.g., *ba ba ba ba*; *di di di di*; *fu fu fu fu*; etc) and 25% were deviant Different sequences, which differed from the Same sequences by ending with a different syllable (AAAB; e.g., *lo lo lo me*). The experiment lasted until 96 additional trials were run (72 standard and 24 deviant trials), until the infant fussed out or until the parent asked to stop the experiment. Two trials were separated by a grey screen displaying only a central blinking cross to attract infants’ gaze. Trials were run in a pseudo-random order, so that two deviant trials were separated by 1 to 6 standard trials. The other half of participants were included in the control group, for whom 50% of trials respected the Same structure (AAAA) and 50% respected the Different structure (AAAB). The experiment lasted until a total of 96 trials were run (48 Same and 48 Different trials), until the infant fussed out or until the parent asked to stop the experiment. Two trials were separated by a grey screen displaying only a central blinking cross to attract infants’ gaze. Trials were run in a pseudo-random order, so that no more than four trials of the same kind could follow each other.

*Experiment 2*: The stimuli and procedure of Experiment 2 were identical to that of Experiment 1, except for one feature: sequences of 5 instead of 4 syllables were used. The unbalanced group heard 75% of standard Same trials (AAAAA) and 25% of deviant Different trials (AAAAB); the control group heard 50% of Same trials (AAAAA) and 50% of Different trials (AAAAB).

*Experiment 3*: The stimuli and procedure of Experiment 3 were identical to that of Experiment 1, except for one feature: sequences of 6 instead of 4 syllables were used. The unbalanced group heard 75% of standard Same trials (AAAAAA) and 25% of deviant Different trials (AAAAAB); the control group heard 50% of Same trials (AAAAAA) and 50% of Different trials (AAAAAB).

*Experiment 4*: The stimuli and procedure of Experiment 4 were identical to that of Experiment 3, except for one feature: in deviant trials, instead of violating the standard structure on the final syllable (AAAAAB in Experiment 3), the structure was violated on the fourth syllable (AAABAA). The unbalanced group heard 75% of standard Same trials (AAAAAA; e.g., *ba ba ba ba ba ba*; *di di di di di di*; *fu fu fu fu fu fu*; etc) and 25% of deviant Different trials (AAABAA; e.g., *lo lo lo me lo lo*); the control group heard 50% of Same trials (AAAAAA) and 50% of Different trials (AAABAA).

*Experiment 5*: The stimuli and procedure of Experiment 5 were identical to that of Experiment 1, except for one feature: in the unbalanced group, instead of being always composed of 4 syllables, standard Same trials were composed of 3 (AAA, 25% of all trials; e.g., *ba ba ba*), 4 (AAAA, 25% of all trials; e.g., *di di di di*) or 5 syllables (AAAAA, 25% of all trials; e.g., *fu fu fu fu fu*). Deviant Different trials were always composed of 4 syllables (AAAB, 25% of all trials; e.g., *lo lo lo me*); the control group heard 50% of Same trials (AAAA) and 50% of Different trials (AAAB). Note that, in the control group, the number of syllables is fixed. The purpose of the control group is to isolate a potential local effect in AAAB sequences. This effect can be measured by comparing responses to AAAB and AAAA sequences, and should be by definition independent from the presence and nature of other sequences.

*Experiment 6*: The stimuli and procedure of Experiment 6 were identical to that of Experiment 5, except for one feature: the number of syllables varied between two and four. Standard Same trials were composed of 2 (AA, 25% of all trials; e.g., *ba ba*), 3 (AAA, 25% of all trials; e.g., *di di di*) or 4 syllables (AAAA, 25% of all trials; e.g., *fu fu fu fu*). Deviant Different trials were always composed of 3 syllables (AAB, 25% of all trials; e.g., *lo lo me*); the control group heard 50% of Same trials (AAA) and 50% of Different trials (AAB).

### Analysis

Fixations were identified by PsyScope X following the dwell-time algorithm (Duchowski, 2007) with the following parameters: WindowLength = 200, MinFixationLength = 100, DistanceFromMean = 0.05. We defined an area of interest (660 pi × 432 pi) corresponding to the surface of the video played on the screen to attract infants’ gaze. The pupil diameter for both eyes was recorded for fixations in that area of interest. For each trial, we considered a baseline time window beginning 500 ms before the onset of the final syllable of the sequence in Experiments 1–3 and 5–6, and 500 ms before the onset of the fourth syllable in Experiment 4. The average pupil diameter in the baseline window was subtracted from all data points.

We excluded trials with less than 75% of pupil diameter information over the entire trial duration (5217 ms) and/or less than 100 ms of pupil diameter information in the baseline time window. The first criterion was chosen so that it would be equally stringent in all experiments; the second criterion ensured there was reliable baseline information in all included trials. Table 2 presents the average number of trials included in the final analyses for each experiment. The first 8 trials in the unbalanced group constituted the familiarization with the standard structure and were not analyzed. Infants with less than 2 good trials per condition were excluded from further analyses. Missing data for good trials were linearly interpolated.

**Table 2.** Average numbers of trials included in the final analyses. Standard deviants from the mean are indicated in parentheses.

**Experiment**	**Group**	**Total #trials**	**#good trials**	**#good same**	**#good different**
Experiment 1 *(4-same)*	Unbalanced 1	49.19 (11.18)	29.62 (13.37)	21.75 (10.90)	7.87 (2.66)
	Control 1	45.12 (23.32)	28.56 (15.06)	14.19 (7.95)	14.37 (8.27)
Experiment 2 *(5-same)*	Unbalanced 2	70.50 (23.56)	40.50 (20.78)	29.75 (15.38)	10.75 (5.74)
	Control 2	64.75 (20.58)	37.94 (22.48)	18.56 (11.62)	19.37 (11.11)
Experiment 3 *(6-same)*	Unbalanced 3	45.19 (20.57)	27.56 (15.24)	20.56 (11.80)	7.00 (3.71)
	Control 3	43.56 (20.07)	26.25 (8.10)	12.94 (4.36)	13.31 (4.16)
Experiment 4 *(4 of 6-same)*	Unbalanced 4	72.44 (18.90)	39.94 (16.57)	30.00 (13.16)	9.94 (3.82)
	Control 4	78.12 (23.29)	44.94 (24.08)	22.19 (12.08)	22.75 (12.19)
Experiment 5 *(all-same)*	Unbalanced 5*	28.56 (12.04)	17.81 (9.26)	8.31* (4.59)	9.50 (5.05)
	Control 5	60.87 (26.77)	34.56 (22.71)	17.37 (11.37)	17.19 (11.54)
Experiment 6 *(all-same)*	Unbalanced 6**	41.56 (9.47)	22.69 (9.55)	10.81** (5.09)	11.87 (4.87)
	Control 6	60.19 (29.29)	30.81 (16.75)	15.25 (8.55)	15.56 (8.39)

* In the Unbalanced group of Experiments 5, only 4-syllable long standard (AAAA) and deviant (AAAB) trials were analyzed.

** In the Unbalanced group of Experiments 6, only 3-syllable long standard (AAA) and deviant (AAB) trials were analyzed.

Previous work using similar paradigms found that the effect of the violation of a sequence structure on pupil dilation typically starts around 1250 ms after the violation onset (and can last up to 1000 ms) (Hochmann & Toro, 2021). We thus ran repeated-measures ANOVAs comparing average pupil dilations in the 1250–2250 ms time window in response to Same and Different sequences in the Unbalanced and Control groups. Bayes factors (BF) were computed using the method in Faulkenberry (2020).

In addition, non-parametrical cluster mass permutation tests (Hochmann & Papeo, 2014; Maris & Oostenveld, 2007) were implemented to probe the variation of pupil dilation in response to Same and Different sequences and the interaction of that effect with the group (Unbalanced or Control).

In Experiments 5–6, because concurrent audio-visual stimulation attracts infants’ attention, which in turn impacts pupil diameters, only those trials with the same number of syllables were comparable. In Experiment 5, we thus only compared Same AAAA and Different AAAB trials. AAA and AAAAA trials were not analyzed. In Experiment 6, we thus only compared Same AAA and Different AAB trials. AA and AAAA trials were not analyzed.

## RESULTS

### Experiment 1: *4-Same*

In Experiment 1, 10- to 12-month-old infants (*N* = 32) were tested on their capacity to detect and generalize the relation *same* between 4 syllables (Same sequences: AAAA; Different sequences: AAAB). Results showed that infants were not surprised by the local change of a syllable in AAAB sequences in the Control group, but were surprised by the change of relational structure in the Unbalanced group, demonstrating that infants could represent the relation *same* between 4 elements. Detailed analyses follow.

For each group (Unbalanced, *N* = 16 and Control, *N* = 16), for each condition (Same and Different Sequence Type), we computed the average pupil dilation between 1250 and 2250 ms after the last syllable onset, which has been identified as the critical interval for the effect of the violation of a sequence structure on pupil dilation (Hochmann & Toro, 2021). A repeated-measures ANOVA revealed no main effect of Sequence Type (Same, Different) (*F*(1, 30) = .28; *P* = .60; *η*^2^ = .009; BF = .21) or Group (Control, Unbalanced) (*F*(1, 30) = 1.49; *P* = .23; *η*^2^ = .047; BF = .38), but identified a significant interaction between the two factors (*F*(1, 30) = 14.12; *P* = .001; *η*^2^ = .320; BF = 84.66). The dilation difference was statistically significant in the Unbalanced group (*M* = .040 mm, *SD* = .050; *t*(15) = 3.19; *P* = .003, one-tail; BF = 19.79), but not in the Control group (*M* = −.030, *SD* = .055; *t*(15) = −2.18; *P* = .98, one-tail; BF = 2.52).

**Figure 1.**
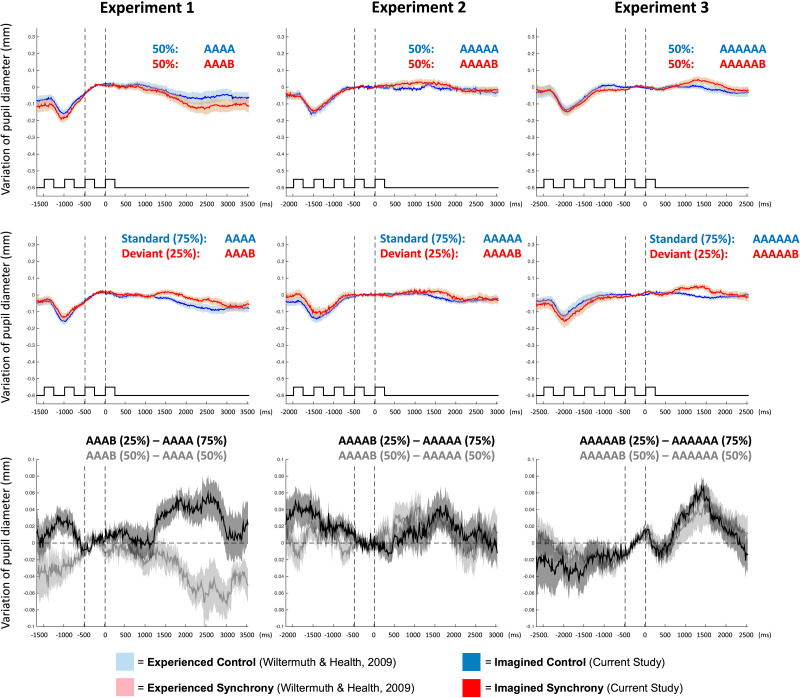
**Results of Experiments 1–3. Variation of pupil diameter in response to sequences of 4 syllables (AAAA or AAAB; Experiment 1; left column), 5 syllables (AAAAA or AAAAB; Experiment 2; middle column) and 6 syllables (AAAAAA or AAAAAB; Experiment 3; right column).** The first row presents the results of the Control groups with equal distributions of same and different sequences. The second row presents the results of the Unbalanced groups with unequal distributions of standard same (75%) and deviant different (25%) sequences. The bottom row presents the comparison of the two groups. The grey curves show the difference between the responses to same and different sequences in the Control group. The black curves show the difference between the responses to standard same and deviant different sequences in the Unbalanced group. On all graphs, light colored areas denote standard errors from the mean.

The analysis of the time-course of pupil dilation with cluster mass permutation tests confirmed these results (Figure 1), finding no significant main effect of Sequence Type (Same, Different), but a significant interaction between Sequence Type and Group (Experimental, Control) in the 1283–2083 ms time window; *P* = .006. The interaction reflected larger pupil dilation for deviant AAAB compared to standard AAAA sequences in the Unbalanced group, but larger pupil dilation for AAAA sequences compared to AAAB sequences in the Control group. Two independent cluster mass permutation tests confirmed this pattern, showing larger pupil dilation in response to deviant AAAB *vs.* standard AAAA sequences between 1350 and 2783 ms (*P* = .02) in the Unbalanced group, and larger pupil dilation in response to AAAA *vs.* AAAB sequences between 1800 and 2533 ms (*P* = .04), and between 2683 and 3500 ms (*P* = .03) in the Control group.

In sum, when infants heard frequent AAAA sequences, they exhibited pupil dilation in response to infrequent deviant AAAB sequences, suggesting either that infants expected only same syllables and were surprised by the presence of a different syllable, or that infants expected exactly four same syllables and were surprised by the absence of the fourth same syllable. In both cases, infants could represent the relation *same* between 4 elements.

### Experiment 2: *5-Same*

In Experiment 2, we tested whether the effect observed in Experiment 1 generalized to relational structures involving five syllables. Two groups of 10- to 12-month-old infants (Unbalanced group: *N* = 16; Control group: *N* = 16) were exposed to Same (AAAAA) and Different sequences (AAAAB). Results showed that infants failed to represent the relation *same* when it involved five elements.

A repeated-measures ANOVA analyzed the average pupil dilations between 1250 and 2250 ms and found no main effect of Sequence Type (Same, Different) (*F*(1, 30) = 3.23; *P* = .082; *η*^2^ = .097; BF = .91), no effect of Group (Control, Unbalanced) (*F*(1, 30) = .34; *P* = .56; *η*^2^ = .011; BF = .21), and no interaction between Sequence Type and Group (*F*(1, 30) = .42; *P* = .52; *η*^2^ = .014; BF = .22). The dilation difference was not statistically significant considering the Unbalanced group alone (*M* = .025 mm; *SD* = .075; *t*(15) = 1.35; *P* = .098, one-tail; BF = .64) or the Control group alone (*M* = .012 mm; *SD* = .036; *t*(15) = 1.34; *P* = .10, one-tail; BF = .63).

Cluster mass permutation tests (Figure 1) analyzing the time-course of pupil dilation found no significant interaction between Sequence Type (Same, Different) and Group (Experimental, Control), and no significant main effect of Sequence Type (*all P*s > .09). Independent cluster mass permutation tests for the Unbalanced and the Control groups found no significant difference between the response to AAAAA and AAAAB sequences (*all P*s > .19).

In sum, the absence of an interaction between Sequence Type and Group supports the conclusion that infants in the Unbalanced group failed to represent the structure of the frequent AAAAA sequences. They did not expect all syllables to be the same.

### Experiment 3: *6-Same*

In Experiment 3, 10- to 12-month-old infants (Unbalanced group: *N* = 16; Control group: *N* = 16) were tested on their capacity to detect and generalize the relation *same* between 6 syllables (Same sequences: AAAAAA; Different sequences: AAAAAB). Results showed that infants failed to represent the relation *same* when it involved six elements. Instead, pupil dilation was evidenced in reaction to the local change of the last syllable in AAAAAB sequences, whether those were infrequent (Unbalanced group) or not (Control group). Detailed analyses follow.

A repeated-measures ANOVA on the average pupil dilation values between 1250 and 2250 ms revealed a main effect of Sequence Type (Same, Different) (*F*(1, 30) = 8.33; *P* = .007; *η*^2^ = .217; BF = 8.91), but no effect of Group (Control, Unbalanced) (*F*(1, 30) = .07; *P* = .80; *η*^2^ = .002; BF = .18), and no interaction between Sequence Type and Group (*F*(1, 30) = .001; *P* = .98; *η*^2^ = .000; BF = .18). Pupil dilatation was larger for Different than Same sequences in both the Unbalanced group (*M* = .033 mm; *SD* = .054; *t*(15) = 2.40; *P* = .015, one-tail; BF = 3.84) and the Control group (*M* = .032 mm; *SD* = .071; *t*(15) = 1.80; *P* = .046, one-tail; BF = 1.28).

Cluster mass permutation tests analyzing the time-course of pupil dilation (Figure 1) confirmed the above results, revealing no significant interaction between Sequence Type (Same, Different) and Group (Unbalanced, Control), but a significant main effect of Sequence Type between 833 and 1900 ms (*P* = .006). Independent cluster mass permutation tests found significantly larger pupil dilation for AAAAAB sequences than for AAAAAA sequences in the Unbalanced group (*N* = 16) between 833 and 1583 ms (*P* = .01), but no significant difference in the Control group (*N* = 16) (all *P*s > .30).

In sum, the absence of an interaction between Sequence Type and Group supports the conclusion that infants in the Unbalanced group failed to represent the structure of AAAAAA sequences in a way that is discriminable from deviant AAAAAB sequences. They did not expect all syllables in the sequence to be the same. The presence of a final different syllable was surprising, but only due to a local effect.

### Effect of the Number of Syllables: Experiments 1, 2, and 3

To confront the results of Experiments 1, 2, and 3, we ran a repeated-measures ANOVA on the average pupil dilation values between 1250 and 2250 ms, with Sequence Type (Same, Different) as within-subject factor and Group (Unbalanced, Control) and Number of Syllables (4, 5, 6) as between-subject factors. The analysis revealed no effect of Group (*F*(1, 90) = .52; *P* = .47; *η*^2^ = .006; BF = .13) or of Number of Syllables (*F*(2, 90) = 2.98; *P* = .056; *η*^2^ = .062; BF = .23), but identified a main effect of Sequence Type (*F*(1, 90) = 9.81; *P* = .002; *η*^2^ = .098; BF = 14.64), a Sequence Type × Group interaction (*F*(1, 90) = 5.46; *P* = .022; *η*^2^ = .057; BF = 1.72) and a three-way interaction between Sequence Type, Group and Number of Syllables (*F*(2, 90) = 3.16; *P* = .047; *η*^2^ = .066; BF = .27). Pairwise *t*-tests showed that pupil dilation for Different compared to Same sequences was larger in the Unbalanced group than in the Control group, when sequences involved four syllables (*t*(30) = 3.76; *P* < .001; BF = 85.20), but not five (*t*(30) = .65; *P* = .52; BF = .22) or six syllables (*t*(30) = .024; *P* = .98; BF = .18). Note that, with respect to the three-way interaction, the frequentist and Bayesian analyses yielded contradictory results. Subsequent analyses nevertheless unambiguously support the conclusion of a stronger global effect with 4 syllables than with 5 or 6 syllables.

For each experiment, for each participant in the Unbalanced group, we computed an index of the global effect (i.e., their success in representing the target structure), comparing the participant’s pupil dilation to the averaged pupil dilation in the control group (in Different compared to Same sequences): for each participant *s*, *I*(*s*) = (Pup_Different_(*s*) − Pup_Same_(*s*)) − Average(Pup_Different_ − Pup_Same_)_ControlGroup_. Pairwise *t*-tests showed that infants performed better when sequences involved four syllables than when they involved five (*t*(30) = 2.50; *P* = .018; BF = 3.65) or six syllables (*t*(30) = 2.64; *P* = .013; BF = 5.00). Performance did not differ when sequences involved five or six syllables (*t*(30) = .32; *P* = .75; BF = .19).

### Comparison of the Local Effect in Experiments 1, 2, and 3

The local effect, isolated in the Control groups, was expected to depend on the number of repeated syllables: the more the A syllable is repeated, the more surprising the B syllable should be. Qualitatively, this was the case. The local effect appeared stronger and more reliable with six syllables (Experiment 3) than with five syllables (Experiment 2) than with four syllables (Experiment 1). A repeated-measures ANOVA analyzed the average pupil dilation in the Control groups, between 1250 and 2250 ms, with Sequence Type (Same, Different) as within-subject factor and Number of Syllables (4, 5, 6) as between-subject factor and found no main effect of Sequence Type (*F*(1, 45) = .35; *P* = .56; *η*^2^ = .008; BF = .17) or Number of Syllables (*F*(2, 45) = 2.52; *P* = .09; *η*^2^ = .101; BF = .27) but a significant interaction between Sequence Type and Number of Syllables (*F*(2, 45) = 5.11; *P* = .01; *η*^2^ = .185; BF = 2.83). Post-hoc *t*-test found that the local effect, measured as the difference of pupil dilation for Different compared to Same sequences, was weaker in Experiment 1 with 4 syllables compared to both Experiment 2 with 5 syllables (*P* = .016) and Experiment 3 with 6 syllables (*P* = .01). The patterns in Experiments 2 and 3, with respectively 5 and 6 syllables, did not differ significantly (*P* = .32). A cluster mass permutation test confirmed these results. It found an effect of Number of Syllables on the local effect in a time window between 1733 and 2367 ms (*P* = .04). In that time window, the local effect differed between sequences of 4 and 5 syllables (AAAB vs. AAAAB; *t*(30) = 2.41; *P* = .02; BF = 3.00) and between sequences of 4 and 6 syllables (AAAB vs. AAAAAB; *t*(30) = 3.07; *P* = .005; BF = 13.99), but not between sequences of 5 and 6 syllables (AAAAB vs. AAAAAB; *t*(30) = 1.16; *P* = .25; BF = .36).

Note that in Experiment 1, we observed a reversed pattern for sequences of 4 syllables in the Control group: dilation was higher for AAAA than for AAAB, despite the two types of sequences being equally frequent. A tentative account for this finding is that the detection of the relation *same* between 4 syllables in AAAA sequences elicits interest that is reflected in larger pupil dilation, whereas the local change in AAAB sequences elicits no pupil dilation (as was already observed in adults; Quirins et al., 2018).

### Experiment 4: *4 of 6-Same*

Experiments 1, 2, and 3 suggest that 10- to 12-month-old infants can represent the relation *same* between a maximum of four elements. To obtain convergent evidence, in Experiment 4, 10- to 12-month-old infants (Unbalanced group: *N* = 16; Control group: *N* = 16) were tested on their capacity to detect and generalize the relation *same* between the first four syllables of a sequence of six syllables (Same sequences: AAAAAA; Different sequences: AAABAA). Results showed that infants could represent the relation *same* between the first four of six elements. Detailed analyses follow.

A repeated-measures ANOVA over the average pupil dilation values between 1250 and 2250 ms showed no effect of Group (Control, Unbalanced) (*F*(1, 30) = 3.36; *P* = .077; *η*^2^ = .101; BF = .97), but a main effect of Sequence Type (Same, Different) (*F*(1, 30) = 7.06; *P* = .013; *η*^2^ = .190; BF = 5.20), and a significant interaction between the two factors (*F*(1, 30) = 6.81; *P* = .014; *η*^2^ = .185; BF = 4.67). The difference in dilation was significant in the Unbalanced group (*M* = .062 mm, *SD* = .077; *t*(15) = 3.25; *P* = .003, one-tail; BF = 22.52), but not in the Control group (*M* = .001 mm, *SD* = .056; *t*(15) = .040; *P* = .48, one-tail; BF = .24).

**Figure 2.**
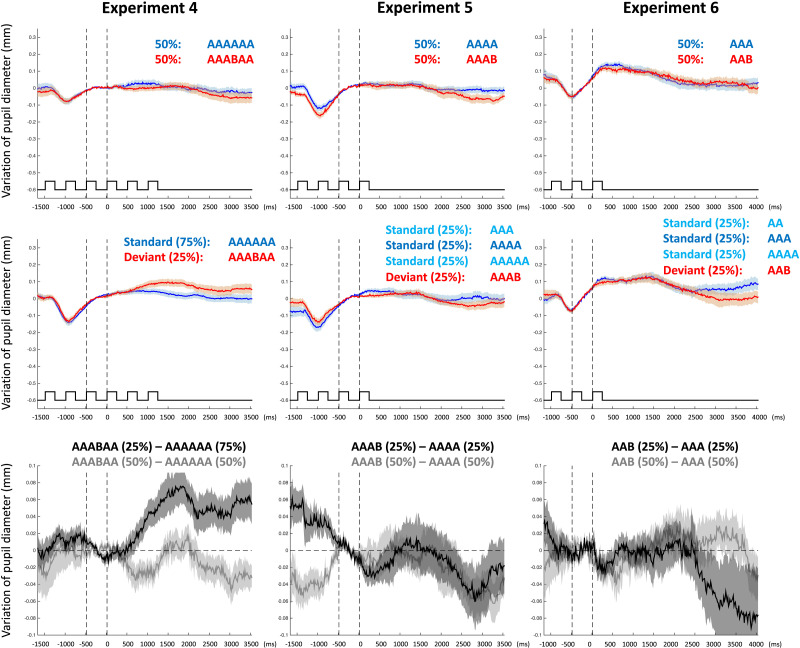
**Results of Experiments 4–6. Variation of pupil diameter in response to AAAAAA and AAABAA sequences (Experiment 4, left column), in response to AAAA and AAAB sequences (Experiment 5, middle column) and in response to AAA and AAB sequences (Experiment 6, right column).** The top row presents the results of the control group with equal distributions of same and different sequences. The second row presents the results of the unbalanced groups with unequal distributions of standard same and deviant different sequences. The bottom row presents the comparison of the two groups. The grey curves show the difference between the responses to same and different sequences in the control group. The black curves show the difference between the responses to standard and deviant sequences in the unbalanced group. On all graphs, light colored areas denote standard error from the mean.

A cluster mass permutation test analyzing the time-course of pupil dilation (Figure 2) confirmed the above results, revealing a main effect of Sequence Type (Same, Different) in the 1283–2083 ms time window; *P* = .05, due to larger pupil dilation for Different sequences compared to Same sequences. This effect was only present in the Unbalanced group, as shown by a significant interaction between Sequence Type and Group (Unbalanced, Control), in the time windows 1000–2100 ms (*P* = .02) and 2167–3500 (*P* = .02). Two independent cluster mass permutation tests confirmed the effect. In the Unbalanced group, we observed larger pupil dilation in response to deviant AAABAA sequences than to standard AAAAAA sequences between 850 and 2167 ms (*P* = .02). No significant difference was found in the Control group at any point in time.

In Experiment 3, infants appeared to have no expectation as to the relational structure involving the sixth syllable of AAAAAA sequences. In Experiment 4 instead, infants exhibited pupil dilation in response to a different syllable in the fourth position, suggesting that infants expected the fourth syllable to be the same as previous syllables. These results thus show that infants can represent the relation *same* between the first four syllables of a longer sequence.

### Experiment 5: *All-Same*

Experiments 2 and 3 suggest that infants cannot represent the relation *same* between five and six elements, respectively. These results also suggest that infants fail at representing *all the same*, which would apply to any sequence of same elements, whether there are four, five or six of them. This observation is consistent with our prediction that infants should fail to disregard the number of entities involved in the relation *same*, as the format of representation that we propose ties together the implicit representations of numerosity and *same*.

To further probe infants’ (in)ability to represent *all the same*, in Experiment 5, we asked whether 10- to 12-month-old infants (Unbalanced group: *N* = 16; Control group: *N* = 16) could detect and generalize the relation *same* between a varying number of syllables (Same sequences: AAA; AAAA or AAAAA; Different sequences: AAAB). Results suggest that they cannot. Detailed analyses follow.

A repeated-measures ANOVA on the average pupil dilation values between 1250 and 2250 ms showed no effect of Sequence Type (Same, Different) (*F*(1, 30) = .12; *P* = .73; *η*^2^ = .004; BF = .19), no effect of Group (Control, Unbalanced) (*F*(1, 30) = .08; *P* = .78; *η*^2^ = .003; BF = .18), and no interaction between Sequence Type and Group (*F*(1, 30) = .035; *P* = .85; *η*^2^ = .001; BF = .18). The difference in dilation was not significant considering the Unbalanced group alone (*M* = −.003 mm; *SD* = .111; *t*(15) = −.11; *P* = .54, one-tail; BF = .24) nor the Control group alone (*M* = −.010 mm; *SD* = .095; *t*(15) = −.41; *P* = .66, one-tail; BF = .27) (Figure 2).

Cluster mass permutation tests analyzing the time-course of pupil dilation (Figure 2) confirmed those results, showing a main effect of Sequence Type between 2400 and 3383 ms (*P* = .04), but no interaction between Sequence Type (Same, Different) and Group (Unbalanced, Control). Both groups showed larger pupil dilation for Same sequences compared to Different sequences, though independent cluster mass permutation tests showed that the difference between Same and Different sequences reached significance for the Control group (between 2500 and 3450 ms, *P* = .05) but not for the Unbalanced group.

Larger pupil dilation for AAAA than for AAAB sequences, already observed in the control group of Experiment 1, may reflect greater attention to the AAAA sequences, possibly because of the local detection of the relation between 4 syllables. In any case, the important finding here is the absence of pupil dilation in response to Deviant AAAB sequences in the mist of AAA, AAAA and AAAAA sequences. In other words, infants did not perceive the structure common to the three types of Same sequences; i.e., all syllables in a sequence are the same. The variability in length of the sequences may have distracted infants, preventing them to extract the common structure. These results nevertheless converge with those of Experiments 2 and 3, where, despite a constant number of syllables, infants failed to realize that all syllables were the same.

Before taking stock, we must address one last issue. Experiment 5 was designed to test infants’ ability to represent *all the same* when the number of syllables vary. The results suggest that infants failed. To represent the sequence structure, they may need to represent the exact number of same elements, which cannot exceed four. However, in the present experiment, one quarter of the sequences, those composed of five syllables, could not be accurately represented (Experiment 2). Can infants represent *all the same*, when each of the sequences can be represented; i.e., when the number of syllables remains below four? In Experiment 6, we tested infants’ ability to represent *all the same*, when the number of syllables varied between two and four.

### Experiment 6: *All-Same*

In Experiment 5, infants failed to see the common structure between AAA, AAAA and AAAAA sequences, suggesting they could not represent *all the same*. Experiment 6 was identical to Experiment 5, except that the number of syllables varied between two and four. We asked whether 10- to 12-month-old infants (Unbalanced group: *N* = 16; Control group: *N* = 16) could detect and generalize the relation *same* between two to four syllables (Same sequences: AA; AAA or AAAA; Different sequences: AAB). Results showed that they cannot. Detailed analyses follow.

A repeated-measures ANOVA on the average pupil dilation values between 1250 and 2250 ms showed no effect of Sequence Type (Same, Different) (*F*(1, 30) = .19; *P* = .66; *η*^2^ = .006; BF = .20), no effect of Group (Control, Unbalanced) (*F*(1, 30) = .70; *P* = .41; *η*^2^ = .023; BF = .26), and no interaction between Sequence Type and Group (*F*(1, 30) = .18; *P* = .67; *η*^2^ = .006; BF = .19). The difference in dilation was not significant considering the Unbalanced group alone (*M* = .0002 mm; *SD* = .092; *t*(15) = .008; *P* = .50, one-tail; BF = .24) nor the Control group alone (*M* = .013 mm; *SD* = .078; *t*(15) = .665; *P* = .26, one-tail; BF = .31).

Cluster mass permutation tests analyzing the time-course of pupil dilation (Figure 2) confirmed those results, showing no main effect of Sequence Type and no interaction between Sequence Type (Same, Different) and Group (Unbalanced, Control). Both groups showed no difference in pupil dilation for Different sequences compared to Same sequences.

The results of Experiment 6 converged with those of Experiment 5, suggesting that infants are unable to detect the common structure between sequences of varying length composed only of repeated syllables, even when the number of syllables remained within the range of infants’ working memory capacity. In sum, infants could not represent *all the same*. As of today, there is little data with respect to infants’ ability to represent a universal quantifier *all* (though see Téglás & Bonatti, 2009; Cesana-Arlotti et al., 2020a). Either such representation and the representation of *same* cannot combine, or infants lack a universal quantifier altogether. The format of representation that we proposed for the relation *same* ties together the relation and the number of entities involved: *two-same* is represented as (*X X*), *three-same* as (*X X X*) and *four-same* as (*X X X X*). In these circumstances, disregarding the number of entities to represent *all-the-same* appears rather difficult.

## DISCUSSION

In six experiments, we investigated whether 10- to 12-month-olds could detect and generalize the structure of syllable sequences, based on the relation *same*. We exposed infants to frequent Same sequences, composed only of identical syllables (e.g., AAAA), and to rare Different sequences ending with (or containing) a different syllable (e.g., AAAB). We analyzed the pupil dilation elicited by Different sequences. Such dilation could reflect a “local effect”, i.e., responding to a local syllable change, and/or, a “global effect”, i.e., responding to the violation of the frequent relational structure of Same sequences. Control groups with equiprobable Same and Different sequences served to isolate the local effects.

In Experiment 1, with 4-syllable-long sequences, pupil dilation was observed in response to rare AAAB sequences. A control group showed that this response could not be accounted for by a local effect, but rather reflected a response to the violation of the AAAA relational structure. Experiment 4 confirmed that infants could represent the relation *same* between four syllables: pupil dilation was observed in response to rare AAABAA sequences and a control group showed that this response could not be accounted for by a local effect.

In Experiments 2, with 5-syllable-long sequences, no pupil dilation could be evidenced. In Experiment 3, with 6-syllable-long sequences, pupil dilation was observed in response to rare AAAAAB sequences, but a control group showed that this response could be accounted for by a local effect alone. Thus, there was no evidence that infants could represent the relational structure of Same sequences, beyond the fourth syllable.

Overall, our results clearly show that infants could represent the abstract relation *same* in sequences of four syllables (Experiment 1), but there is no evidence that they can do so in sequences of five or six syllables (Experiments 2 and 3). This apparent failure is striking when considering that there is more evidence for the relation *same* in sequences of 5 or 6 syllables than in sequences of 4 syllables. Infants could however represent the relation *same* between the first four elements of longer sequences (Experiment 4). The limit of four elements suggests that, in order to represent that sequences are formed of same items, infants hold a representation of each individual item in the relation, and do not rely on quantifiers such as *four*, *six* or *all*. In keeping with this, we found that infants were unable to generalize the relation *same* when the use of a universal quantifier was encouraged, as the number of elements that were *all the same* varied from trial to trial (Experiments 5–6). In sum, infants only succeeded in representing the relation *same*, when the number of items instantiating that relation was constant and within the limit of their working memory capacity.

Our findings thus reveal a discontinuity in the representation of the relation *same*, in the course of cognitive development. Four- to 5-year-old children, as well as adults, represent *same* in a format that can be integrated in a propositional language of thought and combined with quantifiers, generating concepts such as *all the same*, which applies to any set of same individuals, irrespective of cardinality (Hochmann et al., 2017). Such representation is likely to rely on a discrete symbol for the relation *same*, which, in our interpretation is missing in young infants. While the available data suggests that the discontinuity occurs sometime around the fourth year of life, additional work is required to chart a detailed timeline of the development of the representations of *same* and *different*. In particular, the number limit identified here may be even more stringent in younger infants if their working memory is more severely limited (Káldy & Leslie, 2005). Moreover, while the data is so far compatible with the hypothesis that the acquisition of the words “same” and “different” around the fourth year of life plays a determining role in the acquisition of a discrete symbol for the relation *same*, this hypothesis remains to be directly tested.

Challenging our interpretation, one might explain the discontinuity by proposing that infants possess a discrete symbol *S* for *same* but lack quantifiers that would allow representing *all the same* (*all S*). This account predicts that the working memory capacity constrains the number of pairs of same syllables that infants can represent, consistent with what we show in Experiments 1–4. There are reasons, however, to believe that the absence of a discrete symbol *S* for *same* is the most parsimonious account of available data. Considering infants’ failure in Experiment 6 is critical to address this point. We have shown that infants can represent up to 4 same syllables (Experiment 1). Presumably, they can also represent sequences of 2 same syllables and sequences of 3 same syllables. If infants possessed a discrete symbol *S* and use it to represent pairs of same items, they should represent 2 same syllables as *S*, 3 same syllables as *SS* and 4 same syllables as *SSS*. In Experiment 6, infants should thus habituate to the activation of the symbol *S*, or come to expect sequences that activate *S*. Infants should thus react to the different syllable in deviant AAB sequences. In sum, if infants possessed a discrete symbol *S*, we should expect pupil dilation in response to deviant sequences in Experiment 6. But this is not what we observed.

In addition, if infants possessed *S* (but lack *all*) they should succeed in RMTS, where quantifiers play no role. They would be able to represent both AA and BB as *S*, and match *S* to *S*. But we know that children fail at RMTS until the fifth year of life (Hochmann et al., 2017). Finally, the animal literature provides further support to the view that having a discrete symbol *S*—but not representing *all*—is crucial to succeed in RMTS: chimps initially failed at RMTS but can succeed after learning an explicit symbol for same (Thompson et al., 1997). In that study, chimps learned nothing about a universal quantifier *all*. In sum, given the available results from the current and previous studies, the representation of a discrete symbol *S* for *same* is unnecessary to account for infants’ successes, and can hardly accommodate their failures.

We propose that infants’ representation of *same* is built on the aggregation of mental symbols that represent individual entities. These representations of entities cannot be mere perceptual images, as infants are able to generalize the relation *same* to novel (perceptually different) stimuli (here, syllables). We argue that infants represent *two same entities* as (*X X*), where *X* is a variable that refers to a set of properties in the domain under consideration, here the specification of a syllable. Likewise, *three same entities* would be represented as (*X X X*) and *four same entities* as (*X X X X*). Limited working memory capacity makes it impossible to extend this format of representation to *five same entities* or more. In consequence, infants can represent four same entities, but have no expectation about the identity of a fifth or sixth element. Furthermore, those representations of the relation *same* are tight to a specific numerosity (two, three or four), which prevents infants to represent *all the same*, a representation that would disregard numerosity.

In the present work, we used syllables, but the same format of representation is potentially applicable to other modalities and domains of cognition, provided that infants can represent a variable *X* in that domain. Moreover, in our experiments, *same* syllables were identical. Infants may be capable of representing the relation same based on only a subset of the dimensions of a given stimulus. Again, it depends on what type of variables is available to infants. If *X* is a variable for the vowel of a syllable (*X X*) represents the relation *two-same-vowel* (Hochmann et al., 2011; Hochmann et al., 2018a); if *X* is a variable for the shape of a stimulus, (*X X*) represents the relation *two-same-shape* (Hochmann et al., 2018b). Further empirical investigations should generalize our results to other modalities and define the domains in which infants can represent variables.

The finding that the representation of sequences of same elements is limited by working memory capacity calls for the discussion of two findings coming from the research on infants’ working memory. First, while some studies have reported a capacity of four items at 11 months (Ross-Sheehy et al., 2003), most studies report a capacity of about three items (Benavides-Varela & Reoyo-Serrano, 2021; Feigenson & Carey, 2003; Feigenson et al., 2002). Second, when working memory is overloaded, infants sometimes (but not always) exhibit a phenomenon called “catastrophic forgetting”: they remember fewer items than what they are capable of, sometimes even just one (Feigenson & Carey, 2005; vanMarle, 2013; Barner et al., 2007). This happens particularly when infants have to maintain independent representations for objects that have identical features, which resolves into a tendency to erroneously blend those representations (Zosh & Feigenson, 2015).

Although the conditions of our study resemble the circumstances that lead to catastrophic forgetting (the *same* syllables had identical features), infants could encode the first four of six same syllables in Experiment 4. In fact, the detection of identical features triggered the representation of a relational structure, which results in co-dependent entities’ representations. We propose that, whereas catastrophic forgetting may be due to the difficulty to maintain independent representations for each entity, once infants learned the structure (*X X X X*), they did not represent the four identical elements with independent, confusable symbols, but rather represented the second, third and fourth elements as copies of the first element. This results in a reduction of the information load, which can account for the larger working memory capacity observed here (four instead of three items; see also Alvarez & Cavanagh, 2004) and the absence of catastrophic forgetting.

## CONCLUSION

We presented six experiments investigating the representation of the abstract relation *same* in infancy. We hypothesized that infants may not possess a discrete symbol to represent *same* that could be integrated in a propositional language of thought, but rather rely on symbols for the entities involved in the relation. Two predictions followed: infants’ representation of *same* should be constrained by the number of items that they could simultaneously maintain in working memory, and infants should be unable to disregard the number of involved entities, hence failing to represent *all the same*. As predicted, 10- to 12-month-old infants could represent the relation *same* between four, but not more, individual entities. Neither could they represent the relation *same* between a variable number of entities. These results suggest that infant cognition may differ radically from adult cognition in the way it represents abstract relations. While adults possess a propositional language of thought with discrete symbols that refer to abstract relations, infants may rely instead on the juxtaposition of abstract representations of individual entities. This hypothesis, set up here with the investigation of the abstract relation *same*, will be extended to the representation of other abstract relations. More generally, the current research has laid new foundations to studying not only the content of infants’ mental representations, but also their format, in order to eventually characterize precisely not only what infants can think about, but also *how* infants think.

## ACKNOWLEDGMENTS

This work was funded by a Fyssen Foundation Research Grant (2014), the Agence Nationale pour la Recherche grant ANR-16-CE28-0006 TACTIC and the collaborative McDonnell Foundation Grant 220020449. We thank Auriane Couderc, Emilie Serraille and Céline Spriet for their help in recruiting and testing infants; Luca Bonatti, Susan Carey, Nicoló Cesana-Arlotti and Liuba Papeo for comments on previous versions of this manuscript.

## Note

^1^ It is also possible that the role of the word “same” is to make the relation *same* more relevant and more salient. A concept that is verbally labelled may be more likely to be considered in problem solving. Kroupin and Carey (2022a, 2022b) are investigating alternative ways to make the relation more relevant. So far, however, and to the best of our knowledge, there is no evidence that a child ever succeeded at the RMTS without knowing the words “same” and “different”.
